# Virtual Screening, Molecular Dynamics, and Mechanism Study of Homeodomain-Interacting Protein Kinase 2 Inhibitor in Renal Fibroblasts

**DOI:** 10.3390/ph17111420

**Published:** 2024-10-23

**Authors:** Xinlan Hu, Yan Wu, Hanyi Ouyang, Jiayan Wu, Mengmeng Yao, Zhuo Chen, Qianbin Li

**Affiliations:** 1 Department of Medicinal Chemistry, Xiangya School of Pharmaceutical Sciences, Central South University, Changsha 410013, China; 227211035@csu.edu.cn (X.H.); 197211024@csu.edu.cn (Y.W.); 11240811056@stu.ouc.edu.cn (H.O.); 2024103060024@whu.edu.cn (J.W.); 237211014@csu.edu.cn (M.Y.); cz_job@csu.edu.cn (Z.C.); 2 Hunan Key Laboratory of Organ Fibrosis, Changsha 410013, China; 3 Hunan Key Laboratory of Diagnostic and Therapeutic Drug Research for Chronic Diseases, Changsha 410013, China

**Keywords:** renal fibrosis, HIPK2, kinase inhibitor, SIAH2, degradation

## Abstract

**Background/Objectives:** Homeodomain-interacting protein kinase 2 (HIPK2) is critically involved in the progression of renal fibrosis. This study aims to identify and characterize a novel HIPK2 inhibitor, CHR-6494, and investigate its therapeutic potential. **Methods:** Using structure-based virtual screening and molecular dynamics simulations, we identified CHR-6494 as a potent HIPK2 inhibitor with an IC_50_ of 0.97 μM. The effects of CHR-6494 on the phosphorylation of p53 in Normal Rattus norvegicus kidney cells (NRK-49F) induced by transforming growth factor-β (TGF-β) were assessed, along with its impact on TGF-β signaling and downstream profibrotic markers. **Results:** CHR-6494 significantly reduces p53 phosphorylation induced by TGF-β and enhances the interaction between HIPK2 and seven in absentia 2 (SIAH2), facilitating HIPK2 degradation via proteasomal pathways. Both CHR-6494 and Abemaciclib inhibit NRK-49F cell proliferation and migration induced by TGF-β, suppressing TGF-β/Smad3 signaling and decreasing profibrotic markers such as Fibronectin I (FN-I) Collagen I and α-smooth muscle actin (α-SMA). Additionally, these compounds inhibit nuclear factor kappa-B (NF-κB) signaling and reduce inflammatory cytokine expression. **Conclusions:** The study highlights the dual functionality of HIPK2 kinase inhibitors like CHR-6494 and Abemaciclib as promising therapeutic candidates for renal fibrosis and inflammation. The findings provide new insights into HIPK2 inhibition mechanisms and suggest pathways for the design of novel HIPK2 inhibitors in the future.

## 1. Introduction

The global incidence rate of renal fibrosis is steadily increasing, posing an increasingly serious global public health concern [1]. HIPK2 plays a crucial role in modulating fibrosis and inflammation, involving the NF-κB, TGF-β/Smad3, and p53, Wnt/β-catenin pathways [2,3,4,5,6]. HIPK2 exhibits elevated expression in renal models of renal fibrosis and patients with chronic kidney diseases, concurrently modulating fibrosis and inflammatory processes [2,7]. HIPK2 has emerged as a promising therapeutic target for the treatment of fibrosis.

**Figure 1:**
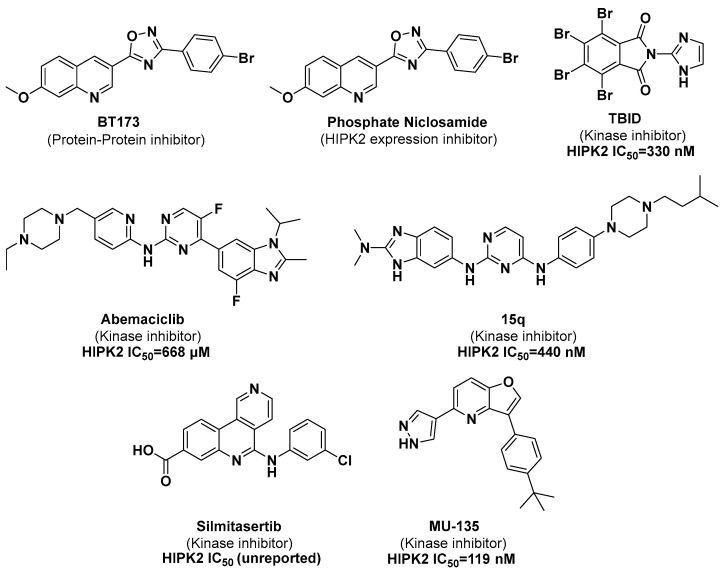
Representative structures of HIPK2 inhibitors.

As shown in Figure 1, BT173 was serendipitously discovered as a protein–protein interaction inhibitor of HIPK2/Smad3, exhibiting moderate inhibitory activity against HIPK2 [8]. As an analogy of BT173, RLA-23174 (structure undisclosed) has entered Phase I clinical trial. This candidate was effective in reducing kidney fibrosis and improving kidney function to significantly extend the survival of Human Immunodeficiency Virus-Associated Nephropathy Transgenic 26 (HIVAN Tg26) and Collagen Type IV Alpha 3 Chain Null Alport Syndrome (Col4a3-null Alport syndrome) mice [9]. Phosphate Niclosamide serves as an inhibitor of HIPK2 expression by disrupting the binding of Smad3 to the promoter sequence of the HIPK2 gene [10]. Kinase inhibitors targeting HIPK2 have been more extensively studied. TBID, a phthalimide derivative, demonstrates moderate inhibitory activity against HIPK2 [11]. Abemaciclib, a cyclin-dependent kinases 4 and 6 (CDK4/6) kinase inhibitor, has been found to exhibit inhibitory effects on HIPK2 kinase activity [12]. The compound **15q**, with structural similarity to Abemaciclib, displays a certain level of enzymatic inhibition against HIPK2 [13]. Additionally, several compounds with HIPK2 kinase inhibitory activity, including Silmitasertib [14] and MU135 [15], have demonstrated nanomolar-level inhibition of HIPK2 kinase. To investigate the role of HIPK2 in renal fibrosis and expand the structural diversity of HIPK2 inhibitors, we conducted a virtual screening of the Topscience compound library (https://www.tsbiochem.com/). Molecular dynamics simulations and kinase inhibition assays show that the screened compound CHR-6494 is a potent HIPK2 kinase inhibitor. Mechanistic validation and phenotyping experiments showed that Abemaciclib and CHR-6494 were not only kinase inhibitors but also promoted ubiquitination of HIPK2 by SIAH2, which led to the downregulation of HIPK2 protein levels and improved fibrosis. This finding may reveal the mechanism of action of the novel HIPK2 kinase inhibitors, which has important research significance.

## 2. Results

### 2.1. Identification of T2476, T16550, T15617, and T9521 Through Virtual Screening

**Figure 2:**
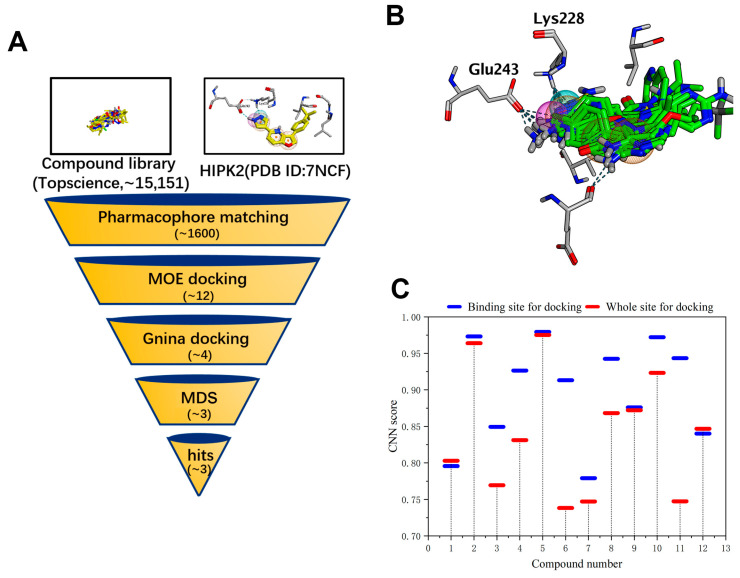
Screening out four compounds in virtual screening. (**A**) Virtual screening flowchart. MDS stands for Molecular Dynamics Simulation. Yellow indicates molecular and grey indicates protein residues. (**B**) After MOE docking screening, 12 compounds were found. Grey indicates protein residues and green indicates 12 compounds. (**C**) The scores for these 12 molecules after docking with Gnina are shown. Blue indicates docking at the ligand binding site while red indicates docking on the entire protein.

**Figure 3:**
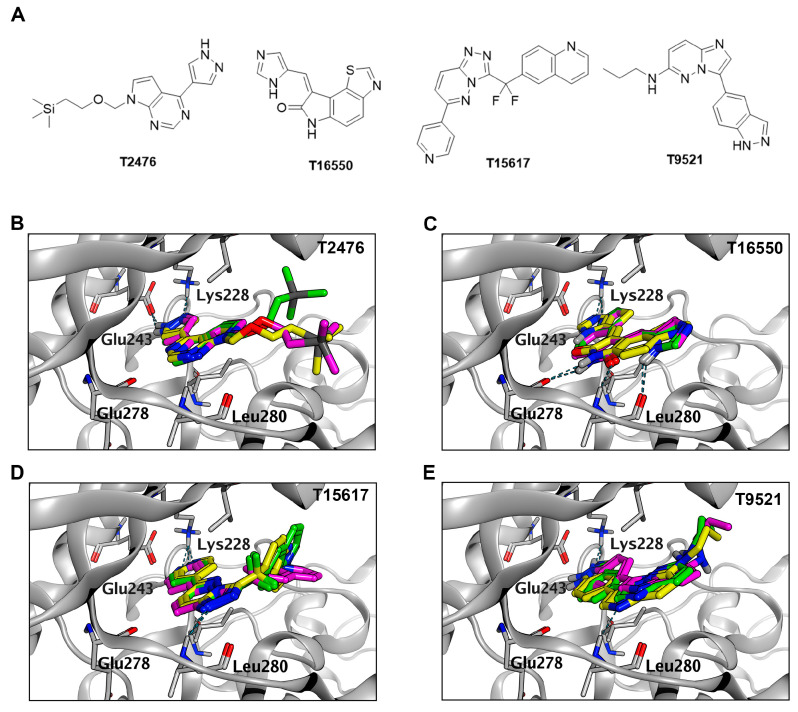
Schematic diagram of molecular docking of the four compounds obtained through virtual screening. (**A**) The structural formulas of the four compounds. (**B**–**E**) represent the docking results for each molecule. Yellow molecules represent the results obtained through MOE docking, green molecules represent docking at the binding site using Gnina, and purple molecules represent docking on the entire protein using Gnina.

The constructed pharmacophore model is depicted in Figure 2A. Employing pharmacophore-based screening of compound libraries led to approximately 1600 compounds, all displaying relevant stereochemical characteristics. Subsequently, these compounds underwent MOE docking with pharmacophore constraints, resulting in 81 compounds that matched both stereochemistry and energy with protein. Following this, a second round of MOE docking was conducted without pharmacophore constraints, resulting in 12 compounds (Figure 2B). In docking at the binding site without constraints on other parameters, all 12 compounds obtained the highest score (Table S1), conforming to pharmacophore features. The final 12 compounds underwent docking using Gnina, targeting both the active site and the entire protein. The obtained results are depicted in Table S1. The CNN score is a value ranging between 0 and 1, utilized to rank the poses of the ligand, with a score of 1 indicating a perfect ligand pose. Poses with higher CNN scores are likely to represent the most stable conformations, with the lowest RMSD values. Specifically, 2 (T2476), 5 (T16550), 8 (T15617), and 10 (T9521) displayed relatively high CNN scores in both dockings (Figure 2C). The CNN score differences for these four compounds between the two docking rounds were relatively small compared to the other eight compounds. Despite T15617 having a slightly larger score difference, we still choose this compound due to its higher docking score at the binding site. Furthermore, the results from docking against the whole protein indicated that they could all return to the ATP competitive binding site, with their poses nearly overlapping those obtained during docking at the binding site (Figure 3).

The docking results of four molecules on MOE and Gnina are depicted in Figure 3. We observed that the conformations of these four molecules remain largely consistent across the three docking scenarios. The pyrazole ring of T2476 interacts via hydrogen bonding with the key amino acids Lys228 and Glu243, with its flexible chain extending into the solvent region (Figure 3B). T16550 exhibits a peculiar behavior where its docking conformations on MOE and Gnina are mutually opposite, displaying centrosymmetric conformations (Figure 3C). According to the docking results from MOE, the nitrogen atom in the imidazole ring interacts with Lys228 through hydrogen bonding, while in the Gnina docking, the thiazole moiety of T16550 extends into the region of key amino acids and interacts with Lys288 via hydrogen bonding. The three docking results of T15617 remain consistent in the key region, where the nitrogen atom of its pyridine ring interacts with Lys228 (Figure 3D). Additionally, its core nitrogen atom also forms a hydrogen bond with Leu280 in the hinge region. Despite slight deviations in the conformations of T9521 among the three docking results, they all interact with the key amino acid Lys288 via hydrogen bonding (Figure 3E). Its core also forms a hydrogen bond interaction with Leu280 in the hinge region. Overall, in the docking results, these four molecules maintain favorable conformations, forming crucial hydrogen bonds and hydrophobic interactions.

### 2.2. Identification of T2476, T16550, and T9521 Through Molecular Dynamics Simulations

The RMSD (root mean square deviation), RMSF (root mean square fluctuation), Rg (radius of gyration), and hydrogen bonds in molecular dynamics simulations partially reflect the stability of protein complexes. Meanwhile, we extracted the conformation of the ligand at the lowest energy state of the complex from the free energy landscape and compared it with docking results obtained from previous virtual screening to assess the molecular affinity.

**Figure 4:**
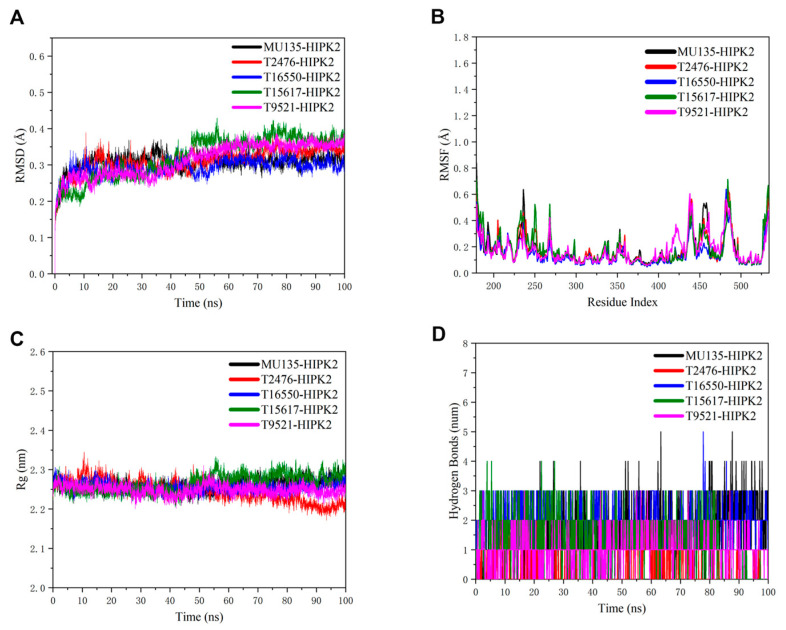
Interaction diagrams derived from 100 ns of MD simulation trajectories, depicting plots of HIPK2 with four compounds. (**A**) The plot of RMSD values over 100 ns for the five complexes. (**B**) The plot of RMSF values over 100 ns for the five complexes. (**C**) The plot of Rg values over 100 ns for the five complexes. (**D**) The plot of hydrogen bond numbers over 100 ns for the five complexes.

Through RMSD analysis, the stability of four protein–ligand complexes was assessed (Figure 4A). The findings reveal that, in comparison to minor fluctuations observed within the initial 40 ns, all complexes stabilized around RMSD values of 0.25–0.35 Å during the last 40 ns, aligning with the behavior of the native ligand. However, T15617 (green color) exhibited a slightly higher RMSD value, indicating that its stability is relatively lower compared to those of the other four small molecules.

Based on the RMSF analysis (Figure 4B), it is apparent that, in comparison to MU135, the RMSF values of the four small molecules remain consistently low throughout the entire simulation process. Moreover, the peak fluctuations observed in these molecules are also below 0.8 in magnitude. This observation suggests the potential for stable binding of these four molecules to HIPK2.

Radius of gyration is also an indicator of the stability of protein complexes, with smaller values indicating a more compact protein structure and a more stable complex. As illustrated in Figure 4C, we found that, compared to MU135-HIPK2, the Rg values of T16550-HIPK2, T15617-HIPK2, and T9521-HIPK2 remained stable between 2.25 and 2.30. Although T2476-HIPK2 exhibited a smaller gyration radius in the last 40 ns, its value was unstable, suggesting that further validation of its stability is required. Based on the comparison of hydrogen bond numbers among the five complexes shown in Figure 4D, we found that the number of hydrogen bonds in T16550-HIPK2, T15617-HIPK2, and MU135-HIPK2 were similar, stabilizing at around 2–3. However, T2476-HIPK2 and T9521-HIPK2 exhibited fewer hydrogen bonds, approximately 1–2. These results indicate that a certain number of hydrogen bonds contribute to the stability of these complexes in the 100 ns molecular dynamics simulations. Nonetheless, hydrogen bond count is not the sole evaluation criterion, and further verification of their stability is required.

**Figure 5:**
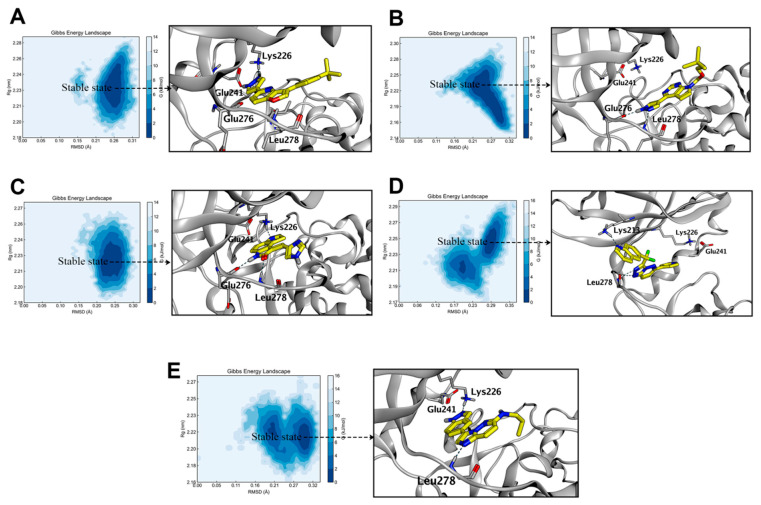
Molecular dynamics simulations, utilizing free energy landscape plots, elucidated the binding modes of HIPK2 with four compounds. The left graphs of (**A**–**E**) ((**A**) MU135-HIPK2, (**B**) T2476-HIPK2, (**C**) T16550-HIPK2, (**D**) T15617-HIPK2, (**E**) T9521-HIPK2) respectively display the free energy landscape plots, with RMSD on the horizontal axis and Rg on the vertical axis. The blue regions represent areas of lower energy, indicating relative stability of the protein complexes. The right graphs of (**A**–**E**) illustrate conformations of the protein–ligand complexes extracted from the lowest energy points on the free energy landscape plots.

To unravel the complex interaction patterns between four molecules and HIPK2, we created the free energy landscape related to their binding (Figure 5) using DuIvyTools v0.5.0 [16]. We used root mean square deviation (RMSD) and radius of gyration as reaction coordinates, applying the Boltzmann distribution formula to convert the energy of each conformation into the corresponding probability. The color intensity in the figure indicates how often each conformation occurs, with darker areas representing lower free energy and lighter areas indicating higher free energy.

We observed that the distribution and extent of the low energy regions for the five protein complexes were roughly similar, although there were some differences in depth. To investigate this, we first identified the conformation with the lowest energy and its corresponding index from the log file of the GROMACS output. Next, we looked up the frame numbers corresponding to this index in the bindex.ndx file, and finally extracted the conformations of the lowest energy complexes based on these frame numbers to confirm whether the position of the ligand within the protein binding pocket aligns with the docking conformations obtained previously.

We found that at the energy minimum, the conformation of MU135 is largely consistent with the reported crystal structure of the complex (Figure 5A and Figure S1). Its pyrazole ring interacts with Lys226 and Glu241 through hydrogen bonding, which also indicates the reliability of our data. The energy-minimized conformation extracted from T2476-HIPK2 (Figure 5B) reveals significant conformational changes in T2476, with some structural elements exposed outside the pocket, lacking crucial interactions, but still exhibiting partial hydrogen bonding and hydrophobic interactions. Therefore, T2476 may still retain some degree of enzymatic inhibitory activity. The conformation of T16550-HIPK2 (Figure 5C) is consistent with the docking results from Gnina, contrary to the docking conformation from MOE, with its thiazole moiety extending towards the critical region. The conformation of T15617-HIPK2 (Figure 5D) is similar to that of T2476-HIPK2, with most of its groups exposed outside the pocket and lacking crucial hydrogen bonding interactions. However, the difference lies in T2476 having better hydrophobic interactions with the loop region, whereas in T15617, the loop region exhibits greater mobility. At this position, lysine interacts with the quinoline ring through hydrogen bonding, thereby obstructing the hydrophobic interaction between the core structure of T15617 and HIPK2. T9521-HIPK2 (Figure 5E) differs from the previous three molecules, as its conformation is largely consistent with the docking results from both MOE and Gnina. The crucial hydrogen bonding and hydrophobic interactions are preserved, demonstrating strong stability. We speculate that T9521 may possess promising inhibitory activity against HIPK2.

Based on the virtual screening and molecular dynamics simulations conducted above, we hypothesize that T9521, T16550, and T2476 may possess potential inhibitory activity against HIPK2, while the inhibitory activity of T15617 is expected to be poor. Therefore, we do not intend to conduct a biological activity test on T15617.

### 2.3. Potential Activity of CHR-6494 (T9521) Identified Through Cell Viability and ADP-GloTM Kinase Assay

We selected Abemaciclib as the positive control drug on ADP-GloTM kinase assay due to its potent inhibition of HIPK2 activity [12]. However, since no mechanism of Abemaciclib in fibrosis and inflammation has been reported in the literature, we will also explore its mechanism in subsequent studies, but will not compare its activity as a positive drug in phenotypic and mechanistic assays. To evaluate the effects of compounds on renal cells in vitro, NRK-49F (Rat renal fibroblast cells) cell lines were cultured and treated with three compounds obtained from our virtual screening. The cell viability was assayed using the Cell Counting Kit-8 (CCK-8) method. As shown in Table 1, T16550 and T9521 showed potent anti-proliferation effect (T16550: IC_50_ = 0.3527 ± 0.00079 μM; T9521: IC_50_ = 3.07 ± 0.32 μM) against NRK-49F compared with T2476 (IC_50_ = 54.89 ± 7.69 μM). In the ADP-Glo™ kinase assay, both compounds exhibited strong inhibitory activity against HIPK2, but T9521 demonstrated a stronger effect (HIPK2 IC_50_ = 0.97 ± 0.04 μM) compared to T16550 (HIPK2 IC_50_ = 3.58 ± 0.06 μM). Given the extremely poor solubility of T16550, we plan to focus further investigation on T9521.

T9521, also known as CHR-6494, is a small-molecule inhibitor of the histone kinase Haspin. There are studies reporting its anti-tumor activity [17], but currently, it seems that there are no relevant literature reports on its role in fibrosis. As shown in Table 1, Abemaciclib and CHR-6494 both exhibit inhibitory effects on TGF-β-induced NRK-49F cells and TNF-α-induced human renal tubular epithelial (HK-2) cells. These findings suggest that these compounds have potential for ameliorating fibrosis and inflammation.

Additionally, we conducted kinase inhibition assays for HIPK1, 2, and 3 with Abemaciclib and CHR-6494 to investigate their selectivity (Table S2). The results showed that at a concentration of 1 μM, both Abemaciclib and CHR-6494 inhibited HIPK2 by over 50% (50.2% for CHR-6494 and 83.2% for Abemaciclib), with Abemaciclib exhibiting a higher inhibition rate. However, at a concentration of 10 μM, Abemaciclib inhibited HIPK2 and HIPK3 by over 90%, whereas CHR-6494 inhibited them by less than 50%. This indicates that although CHR-6494 is less effective than Abemaciclib in inhibiting HIPK2, it demonstrates better selectivity for the HIPK family.

**Figure 6:**
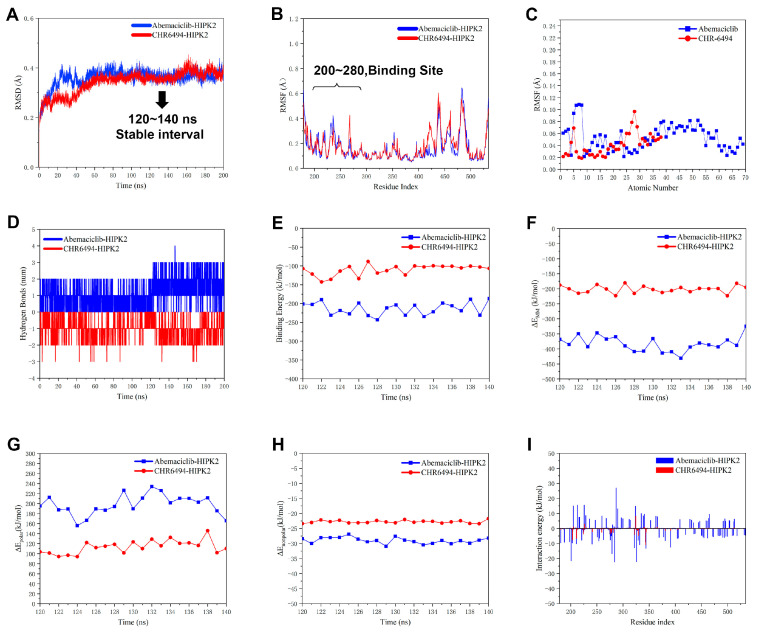
Interaction diagrams derived from 200 ns MD simulation trajectories, depicting plots of HIPK2 with Abemaciclib and CHR-6494. The blue regions represent Abemaciclib–HIPK2, while the red regions represent CHR-6494-HIPK2. (**A**) The plot of RMSD values over 200 ns for Abemaciclib–HIPK2 and CHR-6494-HIPK2. (**B**) The plot of RMSF values over 200 ns for Abemaciclib–HIPK2 and CHR-6494-HIPK2. (**C**) The plot of RMSF values over 200 ns for Abemaciclib and CHR-6494. (**D**) The plot of hydrogen bond numbers over 200 ns for Abemaciclib and CHR-6494 bound to HIPK2. (**E**) The plot of binding energy from 120 to 140 ns for Abemaciclib–HIPK2 and CHR-6494-HIPK2, calculated by MM-PBSA. (**F**) The plot of ΔE_MM_ (the total potential energy of the system) from 120 to 140 ns for Abemaciclib–HIPK2 and CHR-6494-HIPK2. (**G**) The plot of ΔE_polar_ (polar interaction) from 120 to 140 ns for Abemaciclib–HIPK2 and CHR-6494-HIPK2. (**H**) The plot of ΔE_nonpolar_ (nonpolar interaction) values from 120 to 140 ns for Abemaciclib–HIPK2 and CHR-6494-HIPK2. (**I**) The plot of interaction energy from 120 to 140 ns for Abemaciclib–HIPK2 and CHR-6494-HIPK2.

To investigate the differences in activity between CHR-6494 and Abemaciclib, we conducted a 200 ns molecular dynamics simulation using the same protein and parameter settings as before. As shown in Figure 6, the RMSD values (Figure 6A) of Abemaciclib–HIPK2 and CHR6494-HIPK2 remained stable between 0.3 and 0.4 during the latter 100 ns, particularly showing remarkable stability during the 120–140 ns period. The RMSF of amino acid residues (Figure 6B) at the binding site of these two protein complexes are very small, with RMSF values of their ligand (Figure 6C) also less than 0.1, indicating a certain level of stability. By comparing the number of hydrogen bonds (Figure 6D), it was found that the hydrogen bond count of CHR-6494 was inferior to that of Abemaciclib, which may be one of the factors contributing to the difference in activity.

To further explore the differences in activity between them, we determined the binding free energies of the two complexes using the APBS tool [18] in the GROMACS2023 beta suite, following the MM-PBSA (Molecular Mechanics Poisson–Boltzmann Surface Area) method [19,20]. Based on the observation from Figure 6A, we found that the RMSD values of Abemacicilib–HIPK2 and CHR6494-HIPK2 were highly stable and relatively small between 120 ns and 140 ns; therefore, we selected this time period for MM-PBSA calculations and enhanced our operational efficiency by utilizing Jerkwin’s script [20]. The results indicate that the average binding free energy of Abemaciclib–HIPK2 was −163.15 kJ/mol, while that of CHR6494-HIPK2 was −95.40 kJ/mol during the 120 ns to 140 ns period (Figure 6E). The ΔE_MM_ of Abemaciclib–HIPK2 is approximately 150 kJ/mol lower than that of CHR6494-HIPK2 (Figure 6F), indicating a more stable intramolecular interaction for Abemaciclib–HIPK2. The polar solvation energy of Abemaciclib–HIPK2 is higher than that of CHR6494-HIPK2 (Figure 6G), indicating that the Abemaciclib–HIPK2 complex exhibits greater solubility in water and a more stable interaction. The nonpolar interaction between the Abemaciclib–HIPK2 complex and water solvent is slightly stronger than that of CHR6494-HIPK2 (Figure 6H). Based on the analysis of ligand–residue interaction energies (Figure 6I), the interaction between Abemaciclib and HIPK2 residues is more pronounced, resulting in a lower total interaction energy. Additionally, we used MOE to predict several physicochemical properties of CHR-6494 and Abemaciclib, as shown in Table S3. The results indicate that CHR-6494 and Abemaciclib have similar numbers of hydrogen bond donors and acceptors, as well as similar TPSA values, with the main differences being in SlogP values and molecular weight. We hypothesize that the stronger activity of Abemaciclib compared to CHR-6494 may be attributed to its higher SlogP value, suggesting that Abemaciclib has better membrane permeability and stronger hydrophobic interactions with HIPK2.

### 2.4. CHR-6494 and Abemaciclib Suppress Colony Formation and the Migration of TGF-β-Induced NRK-49F Cells

**Figure 7:**
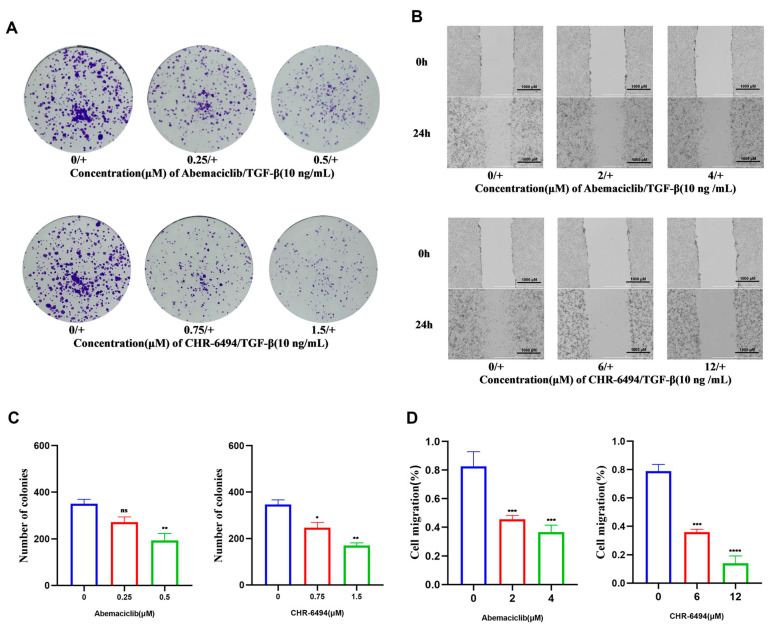
CHR-6494 and Abemaciclib suppress the proliferation and migration of TGF-β-induced NRK-49F cells. (**A**) Representative images of the colony formation assay. (**B**) Representative images of the cell scratch assay. (**C**) Quantitative data analysis of colony numbers for Abemaciclib and CHR-6494 in NRK-49F cells induced by 10 ng/mL of TGF-β. (**D**) Quantitative data analysis of cell migration distance for Abemaciclib and CHR-6494 in NRK-49F cells induced by 10 ng/mL of TGF-β. Data are presented as mean ± SEM, *n* = 3; “ns” stands for no significant difference, **** *p* < 0.0001, *** *p* < 0.001, ** *p* < 0.01, * *p* < 0.05 versus the Control + TGF-β group.

The results of the CCK-8 assay indicate that Abemaciclib and CHR-6494 effectively inhibit the proliferation of NRK-49F cells, which is corroborated by consistent findings in colony formation experiments. Moreover, a concentration-dependent decrease in colony formation is observed with increasing concentrations of Abemaciclib and CHR-6494 (Figure 7A,C). We also assessed the inhibitory impact of both compounds on the migration of NRK-49F cells through scratch assays. The findings underscore the substantial inhibition exerted by Abemaciclib and CHR-6494 on NRK-49F cell migration (Figure 7B). Statistical analysis indicates that Abemaciclib and CHR-6494 inhibit the migration of NRK-49F cells in a concentration- and time-dependent manner (Figure 7D). These phenotypic experiments collectively demonstrate the significant inhibition of fibroblast proliferation and migration by both Abemaciclib and CHR-6494, indicating their considerable potential in the treatment of fibrosis-related diseases.

### 2.5. CHR-6494 and Abemaciclib Inhibit Multiple Profibrotic Signaling Pathways in NRK-49F Cells Treated with TGF-β

**Figure 8:**
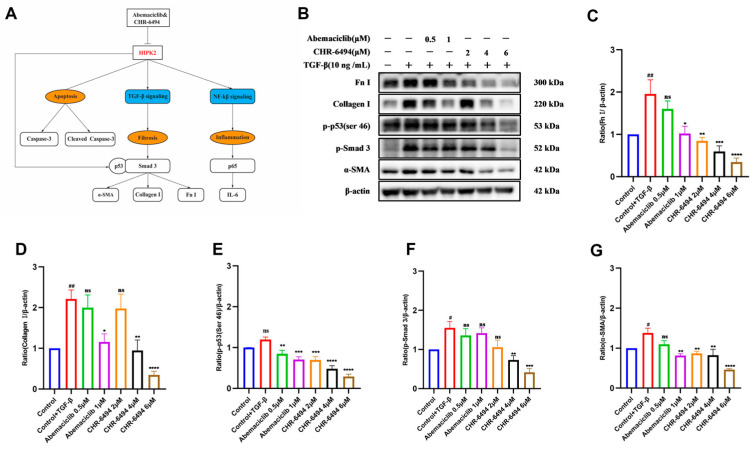
CHR-6494 inhibits multiple profibrotic signaling pathways in NRK-49F cells treated with TGF-β. (**A**) The role of HIPK2 in modulating signaling pathways and associated regulatory factors was investigated. (**B**) The expression levels of Fn-I, Collagen I, p-p53 (Ser46), p-Smad 3, and α-SMA proteins were measured by Western blot analysis. (**C**–**G**) Quantification of the ratios of Fn-I, Collagen I, p-p53 (Ser 46), p-smad 3, and α-SMA normalized to β-actin. Data are presented as mean ± SEM, *n* = 3; “ns” stands for no significant difference, ## *p* < 0.01, # *p* < 0.05 versus the Control group, **** *p* < 0.0001, *** *p* < 0.001, ** *p* < 0.01, * *p* < 0.05 versus the Control + TGF-β group.

As is widely known, HIPK2 is regarded as a crucial regulator of renal fibrosis [20]. HIPK2 plays a key role in activating the p53-mediated TGF-β/Smad3 pathway [21,22], where activation of the TGF-β pathway leads to phosphorylation of its downstream signaling molecule, Smad3, initiating transcriptional activation of fibrosis-related genes such as Collagen I, Fibronectin, and α-Smooth Muscle Actin (α-SMA) [10] (Figure 8A). In normal tissues, Collagen I, Fibronectin, and α-SMA are involved in tissue remodeling and repair [23,24]; however, in fibrotic cells, the expression of these factors is typically significantly increased and closely associated with the progression of fibrosis. When HIPK2 is abnormally expressed, it can activate the TGF-β/Smad3 signaling pathway, further promoting the expression of these factors and thereby driving the fibrotic process. In this study, following validation through colony formation and scratch assays, we confirmed significant inhibition of fibroblast migration and proliferation by both Abemaciclib and CHR-6494. Building upon these findings, we further investigated the potential mechanisms of Abemaciclib and CHR-6494 within the fibrotic pathway. To explore the influence of Abemaciclib and CHR-6494 on renal fibrosis activation in vitro, NRK-49F cells were stimulated with TGF-β (10 ng/mL) and subjected to Western blot (WB) analysis for validation. Initially, the cytotoxicity of Abemaciclib and CHR-6494 was evaluated using a CCK-8 assay. Following a 24 h co-culture with the specified concentrations of compounds, no significant cell death was observed across the concentration gradient (Figure S2A). In this study, given that Abemaciclib exhibited more pronounced toxicity effects in NRK-49F cells and the core objective was to explore the distinct mechanisms of action of Abemaciclib and CHR-6494 rather than a direct comparison of their potencies, we employed a strategy of differential concentration settings based on the IC_50_ values of each compound, rather than using a uniform concentration. This concentration-setting approach was maintained in subsequent experiments to ensure consistency in the study. Subsequently, Western blot experiments were conducted using gradients of 0.5 μM and 1 μM for Abemaciclib, and 2 μM, 4 μM, and 6 μM for CHR-6494. Results and statistical analysis revealed a notable increase in the expression levels of phosphorylated p53 (Ser 46), α-SMA, Collagen I, and Fn-I upon TGF-β stimulation (Figure 8B–G). However, pre-treatment with Abemaciclib and CHR-6494 for 24 h effectively mitigated these alterations. Notably, the changes in expression levels exhibited a dose-dependent reduction with increasing concentrations of Abemaciclib and CHR-6494. Additionally, as depicted in Figure 8B, CHR-6494 exhibited a dose-dependent decrease in phosphorylation levels of Smad3, whereas treatment with Abemaciclib did not yield corresponding changes. These experimental results suggest that CHR-6494 is upregulating fibrosis through the TGF-β/Smad3 pathway.

### 2.6. CHR-6494 and Abemaciclib Mitigate TNF-α-Induced NF-κB Activation in HK-2 Cells

**Figure 9:**
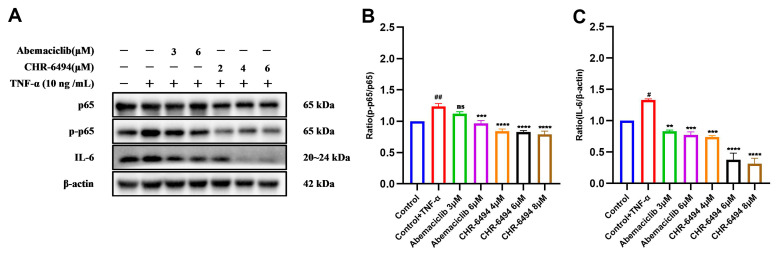
CHR-6494 and Abemaciclib mitigate NF-κB activation in HK-2 cells treated with 10 ng/mL TNF-α for 24 h in vitro. (**A**) The expression levels of p-p65, p65, and IL-6 proteins were measured by Western blot analysis. (**B**) Quantification of the ratios of p-p65, p65, and IL-6 normalized to β-actin. (**C**) Quantification of the ratios of p-p65 normalized to p65. Data are presented as mean ± SEM, *n* = 3; ## *p* < 0.01, # *p* < 0.05 versus the Control group, **** *p* < 0.0001, *** *p* < 0.001, ** *p* < 0.01, versus the Control + TGF-β group.

HIPK2 is a regulatory factor in the NF-κB signaling pathway [6]; consequently, it is imperative to elucidate the impact of Abemaciclib and CHR-6494 on the NF-κB pathway. CCK-8 assay results (Figure S2B) demonstrate that Abemaciclib exhibits no significant cytotoxicity towards HK-2 cells at concentrations of 3 μM and 6 μM, while CHR-6494 shows no significant cytotoxic effects on HK-2 cells at concentrations of 2 μM, 4 μM, and 6 μM. Consequently, these two compounds were utilized at the specified concentration gradients for subsequent Western blot experiments. In this investigation, the efficacies of both compounds against TNF-α-induced NF-κB activation were verified through Western blot analysis. As delineated in Figure 9, treatment of TNF-α-stimulated HK-2 cells with Abemaciclib and CHR-6494 markedly attenuated the expression levels of p-p65 and IL-6. These findings reveal that Abemaciclib and CHR-6494 inhibit the activation of the NF-κB pathway by suppressing HIPK2, highlighting their potential in attenuating TNF-α-induced NF-κB pathway activation, curtailing the expression of the inflammatory mediator IL-6 and mitigating inflammatory responses.

### 2.7. CHR-6494 and Abemaciclib Induce the Degradation of HIPK2 Through the Ubiquitin–Proteasome Pathway

**Figure 10:**
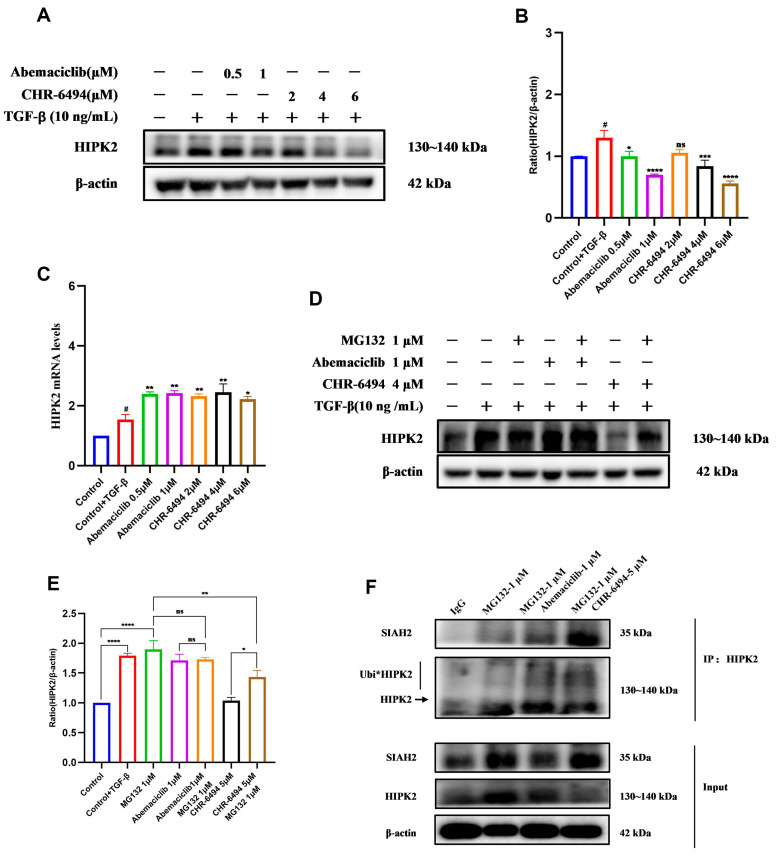
CHR-6494 mitigates the heightened expression of HIPK2 in NRK-49F cells induced by 10 ng/mL of TGF-β for 24 h in vivo. (**A**) The expression levels of HIPK2 proteins were measured by Western blot analysis. (**B**) Quantification of the ratios of HIPK2 normalized to β-actin. Data are presented as mean ± SEM, *n* = 3; “ns” stands for no significant difference, # *p* < 0.05 versus the Control group, **** *p* < 0.0001, *** *p* < 0.001, * *p* < 0.05 versus the Control + TGF-β group. (**C**) The mRNA levels of HIPK2 in the NRK-49F cells were determined by real-time polymerase chain reaction and presented as fold induction over control. Data are presented as mean ± SEM, *n* = 3; “ns” stands for no significant difference, # *p* < 0.05 versus the Control group, ** *p* < 0.01, * *p* < 0.05 versus the Control + TGF-β group. (**D**) The expression levels of HIPK2 proteins were measured by Western blot analysis under the condition of MG132 treatment. (**E**) Quantification of the ratios of HIPK2 normalized to β-actin was performed with MG132 treatment. Data are presented as mean ± SEM, *n* = 3; “ns” stands for no significant difference, **** *p* < 0.0001, ** *p* < 0.01, * *p* < 0.05 versus the Control + TGF-β group. (**F**) Co-IP results indicate that CHR-6494 promotes ubiquitination of HIPK2 in NRK-49F cells. Data are presented as mean ± SEM, *n* = 3.

We unexpectedly discovered that both Abemaciclib and CHR-6494 dose-dependently inhibit HIPK2 protein expression (Figure 10A,B). To investigate the mechanism underlying the downregulation of HIPK2 protein expression, we conducted qPCR experiments. Interestingly, both compounds did not downregulate HIPK2 mRNA levels (Figure 10C); instead, they elicited a slight upregulation, potentially attributed to compensatory feedback mechanisms ensuring adequate cellular response to DNA damage [25,26,27]. Therefore, the reduction in HIPK2 protein expression induced by CHR-6494 and Abemaciclib did not originate at the transcriptional level. The literature shows that SIAH2 is an E3 ubiquitin ligase upstream of HIPK2 [28] and is able to ubiquitinate the degradation of HIPK2. Combining the experimental results with the findings from the literature, we hypothesize that these two compounds may trigger the degradation of HIPK2 protein via the ubiquitin–proteasome system, representing a mechanism of post-translational modification. To further investigate this hypothesis, we employed the proteasome inhibitor MG132 to inhibit HIPK2 degradation, followed by treatment with 5 μM CHR-6494. The results revealed that co-treatment with CHR-6494 and MG132 led to a significant increase in HIPK2 protein expression compared to treatment with CHR-6494 alone (Figure 10D,E). When NRK-49F cells were treated with 1 μM Abemaciclib and MG132, the degradation of HIPK2 was also slightly reversed, but the effect was not significant. When we tried to increase the concentration of Abemaciclib in order to see if this reversal effect would be enhanced, we found that when the concentration of the compound was increased to 2 μM, the cells died in large numbers. We speculated that this might be due to the fact that Abemaciclib is more toxic than CHR-6494; therefore, we did not increase the concentration of Abemaciclib further to explore its effect on degradation. In conclusion, these results suggest that CHR-6494 and Abemaciclib do promote degradation through the ubiquitin–proteasome system.

Given that HIPK2 is regulated by the E3 ubiquitin ligase SIAH2, interaction between SIAH2 and HIPK2 promotes effective polyubiquitination and proteasomal degradation of the kinase [28]. Hence, we conducted co-immunoprecipitation experiments to validate whether Abemaciclib and CHR-6494 mediate proteasomal degradation by promoting SIAH2-mediated ubiquitination of HIPK2. In a fibrosis model induced by 10 ng/mL of TGF-β, treatment with MG132 markedly increased both HIPK2 and SIAH2 expression levels (Figure 10F). Following the subsequent addition of Abemaciclib and CHR-6494, both compounds led to a reduction in HIPK2 protein levels, consistent with previous Western blot experiments. Surprisingly, Abemaciclib also induced a decrease in SIAH2 expression levels, whereas CHR-6494 did not lead to such changes, and the exact mechanism still needs to be further explored. Western blot analysis of HIPK2 from the immunoprecipitation group revealed a decrease in protein levels upon CHR-6494 or Abemaciclib treatment, accompanied by elevated levels of ubiquitinated HIPK2 expression (Figure 10F and Figure S4). Due to the effect of Abemaciclib on SIAH2, we did not test it in the next gene silencing experiments. To further demonstrate that CHR-6494 ubiquitinates HIPK2 degradation by recruiting SIAH2, we treated NRK-49F cells with SIAH2 siRNA to obtain SIAH2-silenced cells. The SIAH2-silenced and normal NRK-49F cells were then treated with 5 μM CHR-6494 and the results showed that the degradation of HIPK2 induced by CHR-6494 was reversed when SIAH2 was silenced (Figure S6). All of the experimental results indicate that CHR-6494 and Abemaciclib enhance the interaction between SIAH2 and HIPK2, thereby promoting the ubiquitination and subsequent degradation of HIPK2.

### 2.8. CHR-6494 and Abemaciclib Enhance TGF-β-Induced Apoptosis in NRK-49F Cells

**Figure 11:**
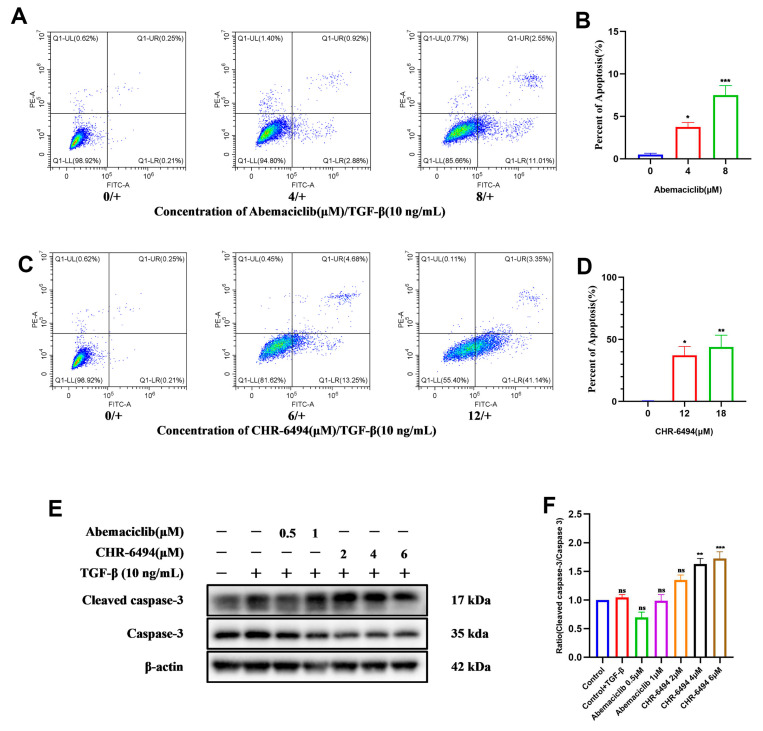
CHR-6494 and Abemaciclib enhance TGF-β-induced apoptosis in NRK-49F cells treated with 10 ng/mL of TGF-β for 24 h in vivo. (**A**,**C**) Scattergram of Abemaciclib and CHR-6494 on the apoptosis and (**B**,**D**) quantitative data analysis of apoptotic NRK-49F. “0/+,4/+,8/+,12/+,18/+” means NRK-49F cells after TGF-β stimulation, treated with different concentrations of Abemaciclib or CHR-6494. (**E**) The expression levels of caspase 3 and cleaved caspase 3 proteins were measured by Western blot analysis. (**F**) Quantification of the ratios of cleaved caspase 3 normalized to caspase 3. Data are presented as mean ± SEM, *n* = 3; “ns” stands for no significant difference, *** *p* < 0.001, ** *p* < 0.01, * *p* < 0.05 versus the Control + TGF-β group.

HIPK2 modulates cellular proliferation, apoptosis, and development by activating the TGF-β/Smad3 signaling pathway mediated by p53 [29]. As a pivotal transcription factor, p53 plays a crucial role in cellular apoptosis [30]. The above experimental results have indicated that the compound CHR-6494 reduces the expression level of p-p53 in NRK-49F cells (Figure 8B) [10]. Building upon these findings, we conducted apoptosis assays and assessed relevant apoptotic markers of Abemaciclib and CHR-6494 in NRK-49F cells. Initially, we used lower concentrations of these two compounds to verify their effects on apoptosis, but the results were not significant (Figure S7). After increasing the concentration, CHR-6494 exhibited dose-dependent promotion of apoptosis in NRK-49F cells (Figure 11C,D). The Western blot results showed a corresponding increase in the expression of the apoptosis marker cleaved caspase 3 (Figure 11E,F). However, the apoptosis effect of Abemaciclib in NRK-49F cells is slight (Figure 11A,B,E,F). Although CHR-6494 and Abemaciclib downregulated the phosphorylation level of p53, it still promoted apoptosis in NRK-49F cells, suggesting that p53 may not act as a pro-apoptotic factor in these cells, but as a pro-fibrotic factor.

## 3. Discussion

Renal fibrosis results in kidney damage and the gradual loss of renal function. Damaged kidney cells and inflammatory cells secrete profibrotic and inflammatory factors, leading to the activation and proliferation of fibroblasts and the production of extracellular matrix (ECM). Despite long-standing efforts to find effective treatments for renal fibrosis, currently available drugs remain very limited. Renal fibrosis is nearly ubiquitous, serving as a common pathway and pathological feature in almost all end-stage renal diseases [31]; therefore, there is an urgent need to identify new therapeutic targets and develop novel treatments for renal fibrosis.

Research findings have elucidated the pivotal role of HIPK2 in the pathogenesis of chronic kidney disease, concurrently modulating key signaling pathways, including NF-κB, TGF-β/Smad3, p53, Wnt/β-catenin, and Notch, all integral to the regulation of fibrosis and inflammation [2,3,4,5,6]. HIPK2 can regulate multiple cellular processes and signaling pathways, thereby influencing cell fate and proliferation. Studies have reported that the role of HIPK2 in cancer is complex, with both tumor-suppressive and oncogenic functions, depending on the specific cellular context [32]. However, the high expression and crucial role of HIPK2 in renal fibrosis have been highly validated in various murine models and patients with chronic kidney diseases (CKDs) [2]. Therefore, inhibition of HIPK2 in these renal fibrosis models may be an effective therapeutic strategy [7].

Currently, research on HIPK2 inhibitors is still in the early stages. BT173 and RLA-23174 are expected to act as PPI inhibitors of HIPK2/Smad3, and Phosphate Niclosamide as an inhibitor of HIPK2 expression. More research on HIPK2 kinase inhibitors should primarily focus on its active site. Given that the crystal structure of a kinase type I inhibitor complex has been reported (PDB ID: 7NCF), we can establish pharmacophores based on the key interactions of MU135 for virtual screening, potentially enhancing the success rate. Compared to high-throughput screening and other biological experimental methods, virtual screening is undoubtedly the best choice for discovering novel HIPK2 kinase inhibitors due to its low cost, short cycle time, and ease of operation.

Most virtual screening processes involve only compound library preprocessing and basic molecular docking, resulting in outcomes that may include molecules in metastable conformations or with multiple isomers, leading to a high false-positive rate. We used MOE for three rounds of screening, including pharmacophore feature matching, pharmacophore-restricted molecular docking, and standard molecular docking. This process rapidly filters out molecular docking results that do not meet the pharmacophore feature. If virtual screening stops at the stage of molecular docking based on the active site, it may yield many inactive molecules, as these molecules may not actually bind to the target site. Therefore, further validation through full protein docking is necessary. We used Gnina [33,34,35,36,37,38,39] for this purpose. Gnina trains convolutional neural network (CNN) models using extensive protein–ligand structure data. These models can more accurately assess the affinity and conformation of molecular docking and generalize well to unseen proteins and ligands. Gnina demonstrates significant advantages in full protein docking. We performed full protein docking and subsequent binding-site docking using Gnina, selecting molecules with high CNN scores in both docking and minimal differences between CNN scores. A large difference between scores may indicate poor binding ability to the target site or a lack of key interactions. This innovative step helped us eliminate some compounds identified through MOE docking. Additionally, we conducted molecular dynamics simulations, comparing the most energetically stable conformations with the initial molecular docking poses. If the variations between them were minimal, we confirmed the molecule as the final selected compound, leading to the discovery of CHR-6494.

The experimental results demonstrated that CHR-6494 and Abemaciclib were able to inhibit the phosphorylation of p53 (Ser 46), which is a direct substrate of HIPK2 [40], thus targeting HIPK2. In addition, CHR-6494 and Abemaciclib were found to be involved in the NF-κB and TGF-β/Smad 3 pathways and to have the ability to inhibit the expression of downstream fibrosis-associated factors, including α-SMA, Fn-I, and collagen I, as well as inflammatory factors, including IL-6. Using kinase activity assay and cellular activity assay, we further determined that CHR-6494 has potent HIPK2 kinase inhibitory activity (IC_50_ = 0.97 ± 0.04 μM) and NRK-49F cellular inhibitory activity (IC_50_ = 3.07 ± 0.32 μM). Meanwhile, we observed that the positive control drug, Abemaciclib, also exhibited potent kinase activity (IC_50_ = 0.45 ± 0.12 μM) and anti-proliferative effects on cells (IC_50_ = 1.59 ± 0.39 μM). Flow cytometry analysis further revealed that CHR-6494 and Abemaciclib promoted early apoptosis in NRK-49F cells induced by 10 ng/mL of TGF-β.

Given that CHR-6494 and Abemaciclib have the ability to reduce HIPK2 protein levels, we investigated the specific mechanism of action. Initially, we conducted qPCR experiments to determine if this effect occurs at the transcriptional level. The results showed that they did not significantly alter the mRNA levels of HIPK2, but instead exhibited a slight upregulation. We then performed degradation experiments, which demonstrated that the downregulation of HIPK2 protein levels was reversed by the addition of the proteasome inhibitor MG132. These experimental results indicated that the downregulation of HIPK2 protein levels was due to the mediation of the proteasome–ubiquitin system. According to the literature, SIAH2 is the upstream E3 ubiquitin ligase of HIPK2, and SIAH2 induces the ubiquitination of HIPK2 [41], leading to its degradation. Therefore, we conducted Co-IP experiments to examine whether they cause the degradation of HIPK2 and whether this is due to the recruitment of the E3 ubiquitin ligase SIAH2. The results were surprising, as they demonstrated that they were indeed able to promote the binding of HIPK2 and SIAH2, leading to the ubiquitinated degradation of HIPK2. In the knockout experiments, silencing SIAH2 reversed the reduction in total protein levels of HIPK2 caused by CHR-6494.

The compounds CHR-6494 and Abemaciclib have shown strong inhibitory activity against kinases and in cells. They have also exhibited dose-dependent inhibition of fibrotic and inflammatory factors, suggesting potential antifibrotic and anti-inflammatory effects in living organisms. Studies have shown that abnormal proliferation and migration of fibroblasts contribute to the accumulation of extracellular matrix, worsening kidney fibrosis [10,42,43]. Our experimental results further demonstrate that CHR-6494 and Abemaciclib inhibit migration and colony formation and promote early apoptosis in TGF-β-induced NRK-49F cells. More importantly, we found that HIPK2 inhibitors may share a common function, which enhances the ubiquitination of HIPK2 by SIAH2, leading to a reduction in total protein levels without significantly affecting HIPK2 mRNA levels. This mechanism may differ from that of PROTACs and molecular glues, which directly recruit SIAH2 to induce HIPK2 degradation. Instead, it may involve an indirect pathway that enhances SIAH2-mediated ubiquitination of HIPK2, though the specific mechanism remains unclear. Literature reports [41] suggest that under abnormal conditions, SIAH2 can enhance the ubiquitination of HIPK2. Therefore, we hypothesize that the effect of HIPK2 inhibitors on promoting HIPK2 ubiquitination may be related to SIAH2. Aside from kinase inhibition, the unique indirect mechanism by which HIPK2 kinase inhibitors promote HIPK2 ubiquitination is quite rare. We believe that such bifunctional HIPK2 inhibitors will have greater therapeutic advantages in clinical treatment in the future.

## 4. Materials and Methods

### 4.1. Protein Preparation and Pharmacophore Modeling and Matching

The crystal structure of human HIPK2 was retrieved from the Protein Data Bank (PDB ID: 7NCF). Next, the QuickPrep tool was utilized to process the protein in MOE (Molecular Operating Environment, the 2022 version). A 3D pharmacophore model for the complex was generated using the pharmacophore elucidation tool. For the virtual screening study, 15,151 bioactive compounds (No. T001) sourced from Topscience (https://www.tsbiochem.com/) were utilized. These compounds were optimized and protonated using MOE. Following preparation, the compound library was screened against pharmacophores using the pharmacophore search tool in MOE2022.

### 4.2. Molecular Docking

Following the matching process, MOE was used to conduct molecular docking. Rigid docking was conducted at the binding site of MU135 while incorporating pharmacophore model constraints. Subsequently, another round of MOE docking was performed using the same parameters as before, except for the exclusion of pharmacophore constraints, based on the results obtained in the preceding step.

Gnina docking (https://github.com/gnina/gnina, accessed on 20 October 2024) was conducted [33,34,35,36,37,38,39] for the compounds obtained from the previous round, employing both ligand-based and whole-protein-based docking approaches (exhaustiveness = 64) and utilizing CNN (Convolutional Neural Network) scoring. Whole-protein docking was utilized to confirm whether the pose with the highest CNN score was located within the ATP binding site. CNN scores tend to be lower compared to sampling within the binding pocket [33]. The difference in the highest CNN scores between ligand-site-based docking and entire-protein-docking was compared to determine whether small molecules show a preference for recognizing the ATP binding site and interacting with the protein in the most probable conformation.

### 4.3. Molecular Dynamics Simulations

The HIPK2 crystal structure obtained from the PDB database has low resolution (2.72 Å), high R-free value (0.251), and numerous residues with errors in electron density (https://www.rcsb.org/3d-view/7NCF?preset=validationReport, accessed on 20 October 2024), which may impact subsequent screening work. Therefore, the AlphaFold2 protein of HIPK2 (UniProt ID: A0A7L0I6C9, https://alphafold.com/) was utilized for molecular dynamics simulations. ORCA5.0 [44] and Multiwfn [45] initially conducted RESP charge calculations and bond optimizations on small molecules. Subsequently, sobtop_1.0 [46] was utilized to create topology files for these small molecules. GROMACS2023 was used to generate topology files for the proteins under the Amber99 force field. Subsequently, the parameters for molecules and proteins were merged. Then, methods previously employed [47] were used as references for subsequent steps. Finally, molecular dynamics simulations were conducted on each system for 100 ns.

### 4.4. Biological Validation Materials

T9521 (3-(1H-indazol-5-yl)-N-propylimidazo[1,2-b]pyridazin-6-amine, CAS ID: 1333377-65-3) was purchased from Shanghai Bide Pharmaceutical Technology Co., Ltd. (Shanghai, China). Abemaciclib Mesylate (LY2835219, CAS ID: 1231930-82-7) was purchased from Shanghai Aladdin Biochemical Technology Co., Ltd. (Shanghai, China).

### 4.5. Cell Culture and Treatment

The normal Rattus norvegicus kidney cell line NRK-49F (CRL-1570) and human renal proximal tubular epithelial cell line HK-2 (CRL-2190) were obtained from ATCC (Mannassas, VA, USA). These cells were cultured in DMEM or DMEM/F12 medium supplemented with 10% or 20% fetal bovine serum (Gibco, Carlsbad, CA, USA), 100 mg/mL of penicillin G, and 100 mg/mL of streptomycin (Gibco, Waltham, MA, USA) under moist conditions at 37 °C and 5% CO_2_.

### 4.6. Kinase Inhibition Assay

The ADP-Glo™ Kinase Assay (#V6930, Promega, Madison, WI, USA) is a luminescent ADP detection assay that provides a universal, homogeneous, high-throughput screening method to measure kinase activity by quantifying the amount of ADP produced during a kinase reaction. In this study, the inhibitory activity of compound T9521 against HIPK2 was assessed using the ADP-Glo™ Kinase Assay following the manufacturer’s standard protocols.

Briefly, the target compound was diluted to the appropriate concentration, and 1 μL was combined with 2 μL of HIPK proteins (8 ng/μL) in an EP tube, followed by centrifugation and incubation at 25 °C for 10 min. Subsequently, 2 μL of a mixture containing 25% 4× Buffer, 5% pre-diluted ATP, 25% MBP, and 45% DDH_2_O was added, mixed, and centrifuged, and then incubated at 25 °C for 1 h. Afterward, 5 μL of pre-melted ADP-Glo™ Reagent was added, mixed, and centrifuged, and then incubated at 25 °C for 40 min. Finally, 10 μL of Kinase Detection Reagent was added to each tube, mixed, and centrifuged, and then incubated at 25 °C for 30 min. Upon completion of incubation, the reaction mixture was promptly transferred to a 384-well plate for fluorescence intensity measurements. Each group included three replicate wells, with 5 μL of reaction mixture added to each well. Fluorescence intensity was measured using a microplate reader (Bio Tek Cytation 5 Cell Imaging Multi-Mode Reader, Winooski, VT, USA), and HIPK activity at each concentration was calculated using the provided formula. IC_50_ values were determined using GraphPad Prism 8 statistical software with nonlinear curve fitting. The formula for kinase inhibition rate is expressed as follows: Kinase Inhibition Rate (%) = (Fluorescence intensity of control group − Fluorescence intensity of experimental group − Background fluorescence intensity)/(Fluorescence intensity of control group − Background fluorescence intensity) × 100%.

### 4.7. Cell Cytotoxicity Assay

Cell viability was evaluated using the CCK-8 assay kit (Dojindo, Kumamoto, Japan). Briefly, cells were seeded into 96-well plates at a density of 3 × 10^3^ cells per well in a final volume of 100 μL and allowed to adhere for 24 h. After 48 h exposure to the respective drugs, the culture medium was substituted with fresh medium supplemented with 10% CCK-8 solution (100 μL per well). The plates were subsequently incubated at 37 °C in the absence of light for 2 h. Absorbance was recorded at 450 nm using a microplate reader (Thermo Fisher Scientific, Waltham, MA, USA) to determine the optical density (OD) values. Cell viability was computed using the following formula: Cell viability (%) = 1 − [(OD of experimental group − OD of blank group)/(OD of control group − OD of blank group)] × 100. The experimental data presented are the means of three independent experiments.

### 4.8. Western Blotting Analysis

NRK-49F and HK-2 cells were seeded into 6-well culture plates at a density of 2 × 10^6^ cells per well. Following a 24 h incubation period, the cells were subjected to treatment with Abemaciclib and the compound CHR-6494 at varying concentrations for an additional 24 h. NRK-49F and HK-2 cells were cultured under stimulation with 10 ng/mL TGF-β and TNF-α, respectively. Subsequently, cell lysis was performed using RIPA cell lysis buffer (Beyotime Biotechnology, Nantung, China) supplemented with protease inhibitors. The appropriate concentration of SDS-PAGE gel, based on the molecular weight of the target proteins, was selected for electrophoresis of denatured proteins in equal amounts, followed by transfer to PVDF membranes (Millipore, Burlington, MA, USA). These membranes were then blocked with 5% non-fat milk in PBST (PBS and 0.1% Tween 20 solution) at room temperature for 2 h. Following blocking, the membranes were incubated overnight at 4 °C with primary antibodies, washed thrice with PBST, and subsequently incubated with HRP-conjugated secondary antibodies (Jackson 111-035-008, West Grove, PA, USA) at room temperature for 2 h. Finally, the target bands were detected using a western fluorescent detection reagent and captured with the imaging system (Bio-Rad, Hercules, CA, USA). The primary antibodies employed in this study are presented in Table 2:

### 4.9. RNA Interference

The siRNA duplexes were produced by Jiangsu Genecefe Biotechnology Co. (Wuxi, China). The sequences of siRNA duplexes are as follows: SIAH2 siRNA, sense 5′-GGAAUCAAUGUCACAAUAUTT-3′, anti-sense 5′-AUAUUGGUGACAUUGAUUCCTT′; negative control (NC) siRNA, sense 5′-UUCUCCGAACGUGUCACGUTT-3′, anti-sense 5′-ACGUGACACGUUCGGAGAATT-3′.

We first inoculated NRK-49F cells uniformly at a density of 2 × 10^6^ cells/well in a 6-well plate and affixed them to the wall for 24 h. The siRNA was transfected with CALNPTM RNAi in vitro transfection reagent (D-Nano Therapeutics, Beijing, China) for 24 h. Then, a specific concentration of the drug was uniformly added to the cells in the 6-well plate and the TGF-β was stimulated for 24 h. After extracting the proteins, the results were detected by the Western blotting method.

### 4.10. Colony Formation Assay

In the colony formation experiment, 1000 cells were seeded into each well of a 6-well culture plate and incubated for 24 h. Following complete adhesion, the NRK-49F cells were stimulated with 10 ng/mL of TGF-β, after which different concentrations of Abemaciclib (0, 0.25, 0.5 μM) and T9521 (0, 0.75, 1.5 μM) were added to each well. NRK-49F cells were then cultured for 1 week under humidified conditions at 37 °C and 5% CO_2_ to allow visible colony formation. Post incubation, colonies were fixed with 4% paraformaldehyde (#BL539A, Biosharp, Hefei, China), stained with 0.05% crystal violet (#G1603, Solarbio, Beijing, China), and analyzed using ImageJ software (https://imagej.net, NIH, Bethesda, MD, USA).

### 4.11. Cell Scratch Assay

For the scratch assay, NRK-49F cells were cultured in 6-well plates at a seeding density of 2 × 10^6^ cells per well. Upon complete cell adhesion and confluency, straight scratch lines were created in each well using a sterile 200 µL pipette tip, followed by PBS washing thrice. Subsequently, cells were stimulated with 10 ng/mL of TGF-β and treated for 24 h with DMEM complete medium containing varying concentrations of Abemaciclib (0 μM, 2 μM, 4 μM) and T9521 (0 μM, 6 μM, 12 μM). Following treatment, images of the same areas as those at the 0 h time point were captured, and cell migration distance was analyzed using ImageJ software. Images at 0 and 24 h were captured using a cell imaging multi-mode reader (Biotek Cytation5).

### 4.12. Apoptosis Assay

For apoptosis experiments, NRK-49F cells were seeded at a density of 2 × 10^6^ cells per well in 6-well culture plates using DMEM medium. Upon reaching full confluence, the cells were stimulated with 10 ng/mL of TGF-β. Subsequently, they were treated with various concentrations of Abemaciclib (0, 4, 8 μM) and T9521 (0, 12, 18 μM) for 24 h. The harvested cells were centrifuged at 1000 rpm for 5 min using a low-temperature centrifuge to remove the culture medium. They were then resuspended in pre-chilled PBS and incubated with a binding buffer containing Annexin V-FITC and propidium iodide (PI) (APExBIO, Houston, TX, USA) for 20 min. The samples were analyzed using a CytoFLEX flow cytometer (Beckman Coulter, Brea, CA, USA).

### 4.13. Real-Time PCR

For the quantitative polymerase chain reaction (qPCR) experiment, we adhered to the standard operating procedures outlined in the kit manual. Total RNA extraction from NRK-49F cells was conducted using the SteadyPure Universal RNA Extraction Kit (Accurate Biology, Changsha, China). Subsequently, the extracted total RNA (400 ng) underwent reverse transcription into cDNA using 5 × Evo M-MLV RT Master Mix (Accurate Biology). The resulting cDNA was directly employed for quantitative PCR analysis, with SYBR Green I fluorescent dye employed for the quantification of target genes. Real-time PCR was executed on the CFX Connect Real-Time PCR Detection System (#1855201, Bio-Rad). The expression level of HIPK2 was determined by RT-qPCR using GAPDH as a reference gene for the 2^−∆∆Ct^ method. The following primer sequences were utilized: Rat GADPH (forward: 5′-TCTCTGCTCCTCCCTGTTCT-3′; reverse: 5′-TACGGCCAAATCCGTTCACA-3′); HIPK2 (forward:5′-CAGTTTGCCCACCAGACCTATAT-3′; reverse5′-CGGGTCCTCCTCCAGTGTTTATA-3′).

### 4.14. Immunoprecipitation with HIPK2

NRK-49F cells were cultured in 10 cm dishes and incubated at 37 °C in a humidified atmosphere for 24 h until they reached confluence. Compounds were diluted with DMEM containing 10 ng/mL of TGF-β to the specified concentrations, with 10 mL added to each dish. After 24 h of drug treatment, cells were harvested and washed with 1 mL of pre-chilled PBS. The cell pellets were collected by centrifugation at 4 °C, followed by resuspension in 200 μL of IP lysis buffer per EP tube and thorough mixing. Cell lysis was achieved using an ultrasonic disruptor, followed by centrifugation at 4 °C to collect the supernatant, which was transferred to new EP tubes. A portion of the supernatant was mixed with 5 × Loading buffer as the Input group. The remaining supernatant was incubated overnight at 4 °C with IP antibodies on a magnetic stirrer. Protein A agarose beads were then added to the EP tubes and incubated for 3 h. After incubation, the samples were centrifuged at low temperature, and the supernatant was discarded. The pellets were washed 2–3 times with PBS and then resuspended in 1 × Loading buffer as the IP group. Following denaturation by heating, the samples were ready for subsequent Western blot analysis.

## 5. Conclusions

In conclusion, through virtual screening, we identified CHR-6494 as a compound with strong HIPK2 kinase activity. Mechanistic validation revealed that both Abemaciclib and CHR-6494 exhibit anti-fibrotic and anti-inflammatory effects. Importantly, our study uncovered a novel mechanism: both Abemaciclib and CHR-6494 promote interaction between SIAH2 and HIPK2, thereby facilitating the ubiquitin-mediated degradation of HIPK2. This finding provides new insights for the design of novel HIPK2 kinase inhibitors in the future.

## Figures and Tables

**Table 1:** Inhibitory activities of compounds inhibiting renal cell proliferation or HIPK2.

Compound	IC_50_ ± SEM (μM)		
	HIPK2	NRK-49F ^a^	HK-2 ^c^
T2476	>100	54.89 ± 7.69	ND ^b^
T16550	3.58 ± 0.06	0.35 ± 0.00079	ND ^b^
T9521	0.97 ± 0.04	3.07 ± 0.32	6.56 ± 0.02
Abemaciclib	0.45 ± 0.12	± 0.39	7.38 ± 0.66

^a^ NRK-49F cells were induced with 10 ng/mL of TGF-β for 48 h. ^b^ Not detection. ^c^ HK-2 cells were induced with 10 ng/mL of TNF-α for 48 h.

**Table 2:** All monoclonal antibodies covered in this article.

Antibody Name	Antibody Branding/Item Number	Source
β-actin	Proteintech/# 20536-1-AP	China
HIPK2	Proteintech/# 55408-1-AP	China
a-SMA	CST/# 19245	USA
Fibronectin	CST/# 26836	USA
Collagen I	CST/# 72026	USA
Caspase-3	CST/# 9662	USA
Cleaved-caspase-3	CST/# 9661	USA
IL-6	CST/# 12153	USA
P-p53 (Ser46)	CST/# 2521	USA
P53	CST/# 2527	USA
P-Smad3	CST/# 9520	USA
Smad3	CST/# 9523	USA
P-p65	CST/# 3033	USA
P65	CST/# 8242	USA
SIAH2	Proteintech/# 12651-1-AP	China

## Data Availability

The data presented in this study are available in Supplementary Materials. The raw data supporting the conclusions of this article will be made available by the authors on request.

## Supplementary Materials

The following supporting information can be downloaded at: https://www.mdpi.com/article/10.3390/ph17111420/s1, Table S1: Docking scores of 12 compounds in MOE and Gnina; Table S2: Inhibitory activity of target compounds against three sub-types of HIPKs; Table S3: Physicochemical properties of CHR-6494 and Abemaciclib; Figure S1: Schematic representation of the overlay between the HIPK2-MU135 crystal structure and the lowest energy conformation of the HIPK2 (Alpha-fold-predicted protein)-MU135 complex extracted from molecular dynamics simulations; Figure S2: CCK-8 assay; Figure S3: Expression levels of total p53 proteins were measured by Western blot analysis; Figure S4: Ubiquitinated HIPK2 protein bands obtained by incubation with ubiquitin antibody in Co-IP experiments; Figure S5: NRK-49F cells treated with three different SIAH2 siRNAs; Figure S6: NRK-49F cells treated with CHR-6494 in the presence or absence of SIAH2 siRNAs. Figure S7: Abemaciclib and CHR-6494 promote TGF-β-induced apoptosis in NRK-49F cells induced by 10 ng/mL of TGF-β for 24 h.
