# Supplementary material for: Virtual Screening, Molecular Dynamics, and Mechanism Study of Homeodomain-Interacting Protein Kinase 2 Inhibitor in Renal Fibroblasts
Source: Pharmaceuticals (Basel). 2024 Oct 23;17(11):1420. doi: 10.3390/ph17111420 (PMC11597050; doi:10.3390/ph17111420)
Supplement: Supplementary file 1 [file pharmaceuticals-17-01420-s001.zip › pharmaceuticals-3242140-supplementary.pdf]

Virtual Screening, Molecular Dynamics, and Mechanism Study of Homeodomain-Interacting Protein Kinase 2 Inhibitor in Renal Fibroblasts

Xinlan Hu, Yan Wu, Hanyi Ouyang, Jiayan Wu, Mengmeng Yao, Zhuo Chen and Qianbin Li

Table of contents:

Table S1. Docking scores of 12 compounds in MOE and Gnina

No	No	Topscience ID	Structure	GBVI/WSA score（kcal/mol)	Gnina CNN score	
					ligand site docking	whole site docking
1		T1838	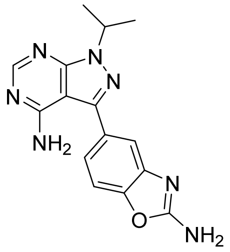	-6.9796	0.7956	0.80292
2		T2476	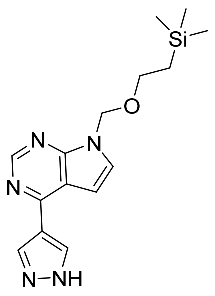	-7.8758	0.97321	0.96391
3		T2477	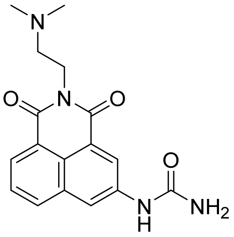	-7.4859	0.84939	0.76942

4	T4410	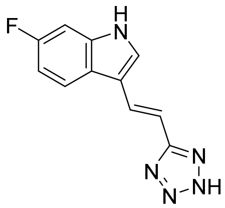	-5.8651	0.92651	0.83127
5	T16550	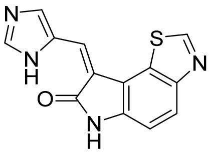	-6.6839	0.97941	0.97519
6	T23176	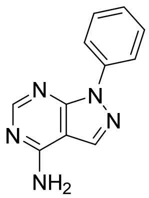	-6.4680	0.91319	0.73824
7	T8987	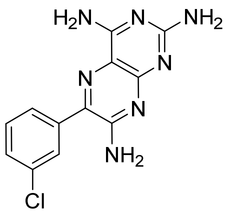	-6.6209	0.77914	0.74716
8	T15617	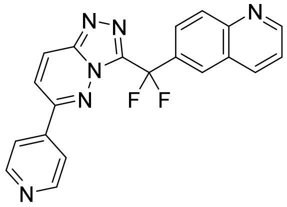	-7.8276	0.94256	0.86828
9	T11942	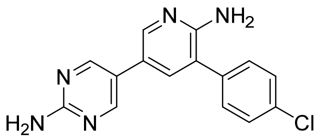	-6.3477	0.87604	0.87222
10	T9521	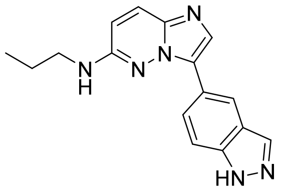	-7.5077	0.97211	0.92335
11	T37292	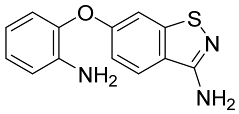	-6.2505	0.9434	0.74737
12	T60181	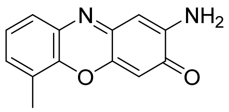	-6.7080	0.84011	0.84673

Table S2. Inhibitory activity of target compounds against three sub-types of HIPKs

Compound	IR[a]<br>（HIPK2[b]，%）	IR<br>（HIPK1[c]，%）	IR<br>（HIPK3[c]，%）
Abemaciclib	83.20	91.25	97.45
CHR-6494	50.20	47.78	36.33

[a]IR: inhibition rate; [b] inhibition rate at 1 μM; [c] inhibition rate at 10 μM.

Table S3. Physicochemical properties of CHR-6494 and Abemaciclib

Name	Structure	Acceptor Count	Donor Count	TPSA[a]	SlogP[b]	MW[c]
CHR-6494	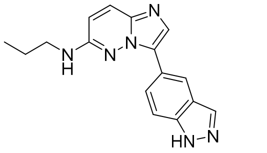	6	2	70.89	2.61	292.35
Abemaciclib	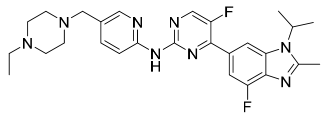	8	1	75.00	5.30	506.61

All these properties were predicted using MOE2022. [a] TPSA (Topological Polar Surface Area) is an indicator of the polar surface area of a molecule, used to predict drug bioavailability and cell membrane permeability. [b] SlogP in MOE is a calculated descriptor representing a molecule's hydrophobicity, predicting its logP value based on molecular structure and properties. [c] Molecular Weight

Figure of contents:

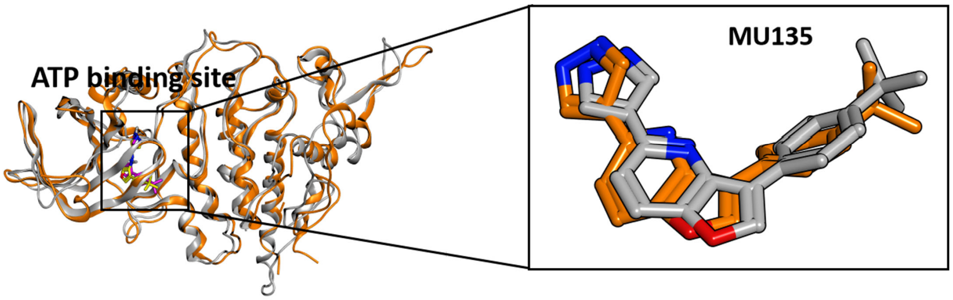

Figure S1. Schematic representation of the overlay between the HIPK2-MU135 crystal structure and the lowest energy conformation of the HIPK2 (Alphafold-predicted protein)-MU135 complex extracted from molecular dynamics simulations. The white part represents the HIPK2-MU135 crystal structure; The orange part represents HIPK2 (Alphafold-predicted protein)-MU135.

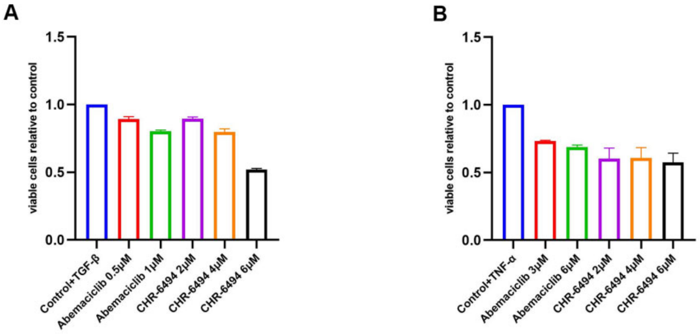

Figure S2. CCK-8 assay. (A) NRK-49F cells were treated with indicated amount of CHR-6494 and Abemaciclib for 24h. (B) HK-2 cells were treated with indicated amount of CHR-6494 and Abemaciclib for 24h. Data are expressed as mean ±SEM of three independent experiments.

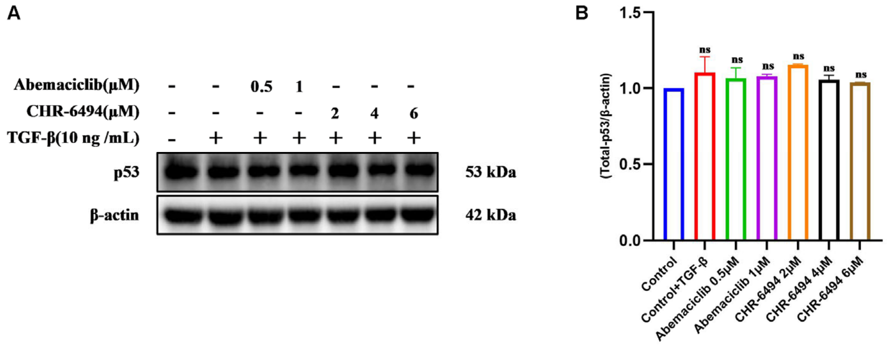

Figure S3. The expression levels of Total-P53 proteins were measured by western blot analysis. (A) The expression levels of Total-P53 proteins were measured by western blot analysis. (B) Quantification of the ratios of Total-P53 normalized to β-actin. Data were presented as mean ± SEM, n=3; "ns" stands for no significant difference.

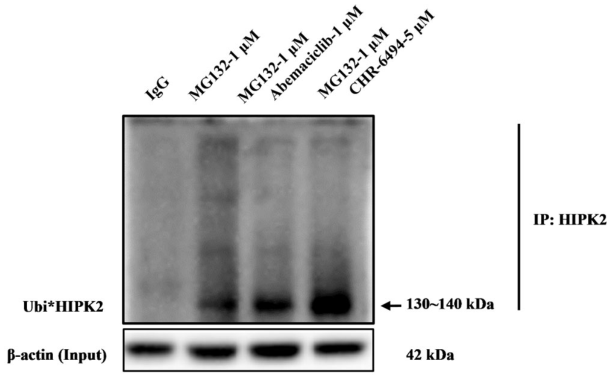

Figure S4 Ubiquitinated HIPK2 protein bands obtained by incubation with ubiquitin antibody in Co-IP experiments.

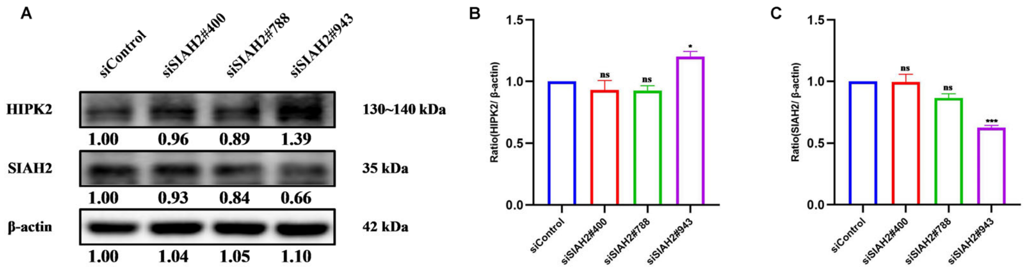

Figure S5 NRK-49F cells were treated with three different SIAH2 siRNAs. (A) NRK-49F cells were treated with three different SIAH2 siRNAs, followed by Western blot analysis. (B, C) Expression of HIPK2 and SIAH2 in SIAH2 siRNA-treated NRK-49F cells. Data were presented as mean ± SEM, n=3; "ns" stands for no significant difference, ***P < 0.001, *P < 0.05 versus the siControl group.

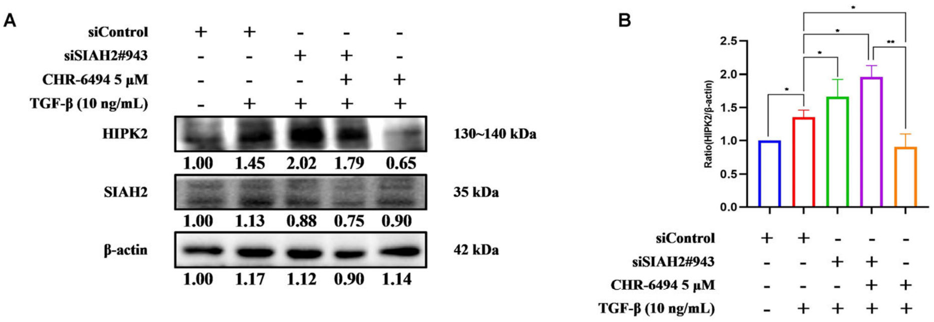

Figure S6. NRK-49F cells were treated with CHR-6494 in the presence or absence of SIAH2 siRNAs#943. (A) NRK-49F cells were treated with CHR-6494 in the presence or absence of SIAH2 siRNAs#943, followed by Western blot analysis. (B) Expression of HIPK2 protein. Data were presented as mean ± SEM, n=3; **P < 0.01, *P < 0.05 versus the corresponding control group.

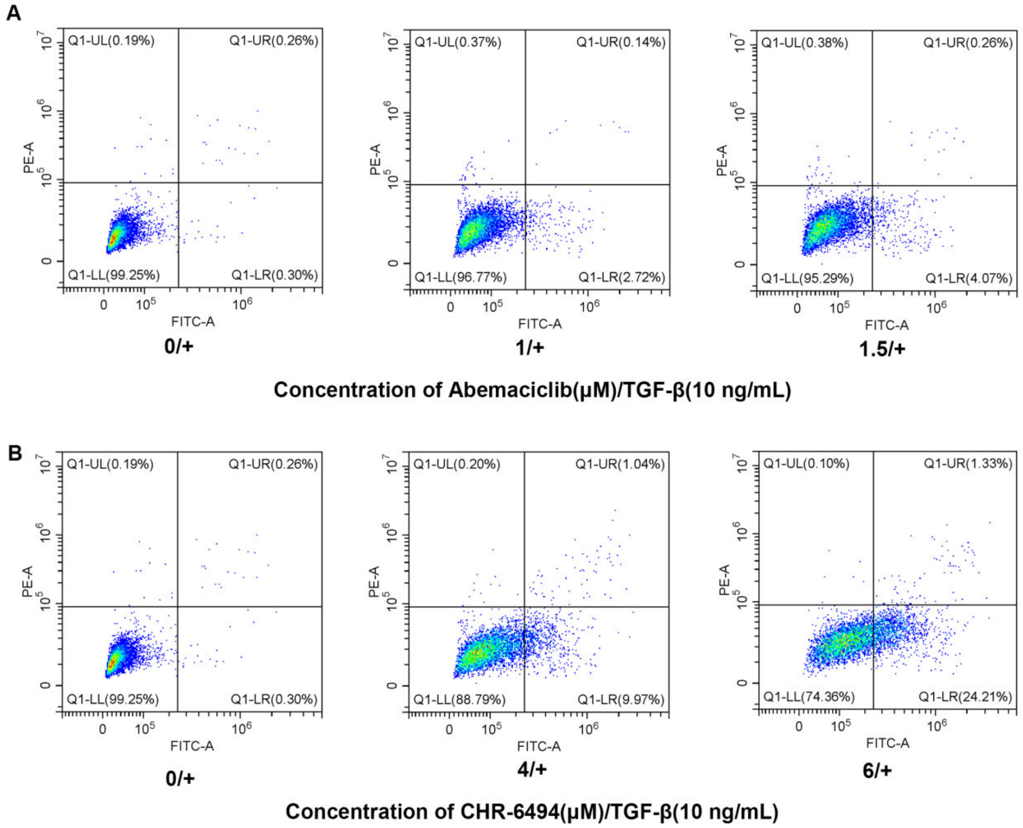

Figure S7. Abemaciclib and CHR-6494 promote TGF-β-induced apoptosis in NRK-49F cells induced by 10 ng/mL of TGF-β for 24 hours. (A) Scattergram of Abemaciclib on the apoptosis. (B) Scattergram of CHR-6494 on the apoptosis. Data were presented as mean ± SEM of three independent experiments.
